# Online lessons of human anatomy: Experiences during the COVID‐19 pandemic

**DOI:** 10.1002/ca.23805

**Published:** 2021-11-01

**Authors:** Daniela Zarcone, Daniele Saverino

**Affiliations:** ^1^ Department of Experimental Medicine University of Genoa Genoa Italy; ^2^ Ospedale Policlinico San Martino Genoa Italy

**Keywords:** human anatomy, medical education, medical student

## Abstract

The social distancing measures necessitated by the COVID‐19 pandemic have resulted in the migration of human anatomy lessons to virtual platforms. Even student communities have had to relocate online. The virtual replacement of visual–spatial and social elements, essential for studying anatomy, has posed particular challenges for educators. Our department used Microsoft Teams, an online communication platform, in conjunction with Visible Body, a 3D anatomical modeling program, EdiErmes online resources, and Leica Acquire for teaching microscopic anatomy. We delivered about 160 h of both synchronous and asynchronous lessons for students on the medical degree program per academic year. In this study, we compare face‐to‐face and distance teaching in order to define these different approaches better and to evaluate the final student scores. The aim is to debate the relevance of distance learning pedagogy to the design of new online anatomy teaching courses and the development of online learning. Analysis of the final scores showed that anatomy examinations after the online course had a statistically significantly higher average value than those obtained at the end of the face‐to‐face course. The experience at the University of Genoa shows that distance learning in the teaching of human anatomy was perceived by most students as useful and positive. Distance learning can be an effective support for anatomy teaching, facilitating a different mode of learning in which lessons and study are more sensitive to the individual's schedule and needs. Of course, we should not and cannot exclude face‐to‐face teaching.

## 1 INTRODUCTION

On 30 January, the World Health Organization declared COVID‐19 a global health emergency (Sohrabi et al., 2020). On March 6, 2020, the Italian Government decided on a national lockdown because of the COVID‐19 pandemic, which stopped face‐to‐face teaching (F2F) in schools of all levels, including universities (DPCM 8‐3‐2020 GU n. 59 8‐3‐2020, 2020). Over the next few days, universities around the world virtually stopped giving F2F lessons. The different faculties, not only in Italy, have therefore sought to develop remote and/or virtual alternatives in order to continue teaching (Evans et al., 2020). Universities, including the one in Genoa, were immediately organized to provide intensive courses to allow teachers to arrange their various courses online, assuming—as then happened—that this method would continue throughout the academic year 2020/2021.

The use of virtual resources in anatomy teaching has grown significantly over the past two decades (Allsop et al., 2020; Grainger et al., 2020; Trelease, 2015). This is because in many universities around the world, including our own, anatomy is not taught using cadaveric material in practical classes and dissections (Saverino, 2020). Before the pandemic, anatomy teaching involved F2F lessons based on the projection of images and films of human dissection, and practical lessons using the microscope to study microanatomy. Therefore, while it was relatively easy to replace live lessons with lessons recorded both synchronously and asynchronously, it was more complicated to organize practical lectures on microanatomy online. Also, it was less clear how online learning could replace practical lessons, especially in respect of final student scores.

During the anatomy course, the faculty of medicine had to overcome problems related to a reduction of visual–spatial learning, a loss of student to student and student to teacher interactions, and a continuous involvement of students with virtual resources.

The University of Genoa responded to the interruption of F2F lessons by increasing the availability of asynchronous resources. These included EdiErmes online resources (Milano, Italy), Acland's Human Anatomy Video Atlas (Lippincott Williams & Wilkins, PA, USA) and 3D‐modeling programs such as Visible Body (Argosy Publishing Inc., MA, USA), which seemed to be working (Byrnes et al., 2021; Evans et al., 2020; Iwanaga et al., 2021; Longhurst et al., 2020; Moszkowicz et al., 2020). In addition, synchronous webinars were implemented using Microsoft Teams (Microsoft 365, WA, USA).

Our department implemented virtual microanatomy applications for medical students for about 2 months during the lockdown using “Leica Acquire, version 3.4.6” (Leica Microsystems, Switzerland). Its use as a virtual classroom for pathological anatomy activities was demonstrated previously (Attardi et al., 2018; Hang et al., 2015). This platform now offers functions with great interactivity and collaborative potential; however, its use has not yet been described in anatomy education.

This period was characterized by serious concerns among anatomy teachers, especially about the loss of F2F practicals. Anatomy teachers have faced several challenges including time, resources, technical skills, and educational innovation, which will continue to support the learning of our students during the COVID‐19 emergency period. However, these challenges could transform into an opportunity to improve the quality of our current F2F teaching when the situation returns to normal (Iwanaga et al., 2021).

Regarding the human anatomy course in the Medicine and Surgery undergraduate course at the University of Genoa, the COVID‐19 pandemic has made it possible to compare F2F teaching with online teaching in the same first‐year and second‐semester undergraduate students over two academic years. In this article, we describe our approach to online anatomy training activities during lockdown to improve student–student and student–teacher interactions and to foster a virtual learning community. We also compare the scores in human anatomy examinations between students who have received F2F lessons and online lessons.

## 2 MATERIALS AND METHODS

### 2.1 Course organization

The student cohorts included first year medical students. Lecture topics during the pandemic paralleled those that would have been addressed during canceled F2Fs and included topographic anatomy, visceral anatomy (cardiovascular, lymphatic, respiratory, urinary, gastrointestinal, endocrine, genital, and tegumentary systems), and microanatomy. The online teaching was designed to remain in line with the traditional F2F medical curriculum.

Lessons were scheduled to last 100 min, reflecting the time allocated to equivalent F2F theoretical and practical classes. The presentations were constructed using PowerPoint (Microsoft 365, WA) and addressed a standardized set of learning outcomes while allowing for the tailoring of content to specific group needs. Slides included traditional text and diagrams, but emphasis was placed on incorporating clinical, radiological, and cadaveric material wherever possible.

At the end of the semester, the students took practical and oral examinations. The assessments were conducted by the same teachers. Marks/scores were established by the Academic Rules and Regulation for Italian Universities (the minimum pass score is 18/30, while the maximum is 30/30 cum laude). For the theoretical lessons, we compared the final scores between two different academic years: 2018/2019 (249 students, 164 females and 85 males; 13 international students), who received F2F lessons; and 2019/2020 (248 students, 166 females and 82 males; 19 international students), who received online lessons. For the microanatomy laboratory, students were distributed nonrandomly into groups of 18–20. Each group was allocated the same teacher for each session to guarantee continuity. Each webinar focused on a slideshow presentation developed and uploaded by faculty members to the Teams platform.

### 2.2 Communication during lessons

Microsoft Teams can facilitate bidirectional flow of written, oral, and visual communication. In our asynchronous lessons or during the “Question Time,” tutors communicated with the students by audio‐visual means. The students were allowed to interact in whichever way they found most comfortable, which included writing or drawing on the slides, typing into an instant messaging chat bar, or using their microphones. In order to preclude background noise, students were asked to mute their microphones and unmute as needed.

**FIGURE 1:**
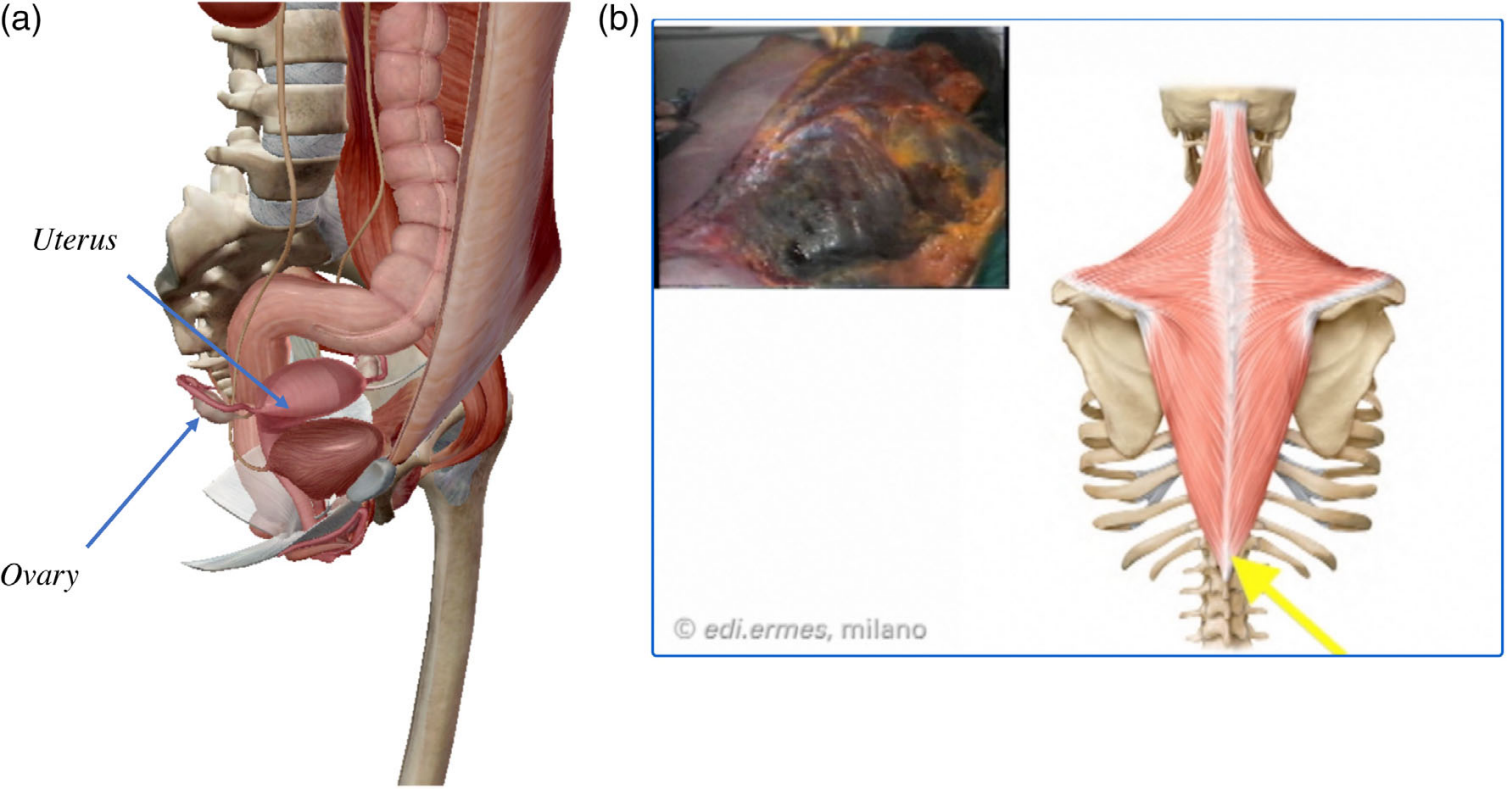
Examples of broadcasting by visible body 3D models (a) and dissection clips (b)

Faculty members guided the students through the slideshow content, which incorporated demonstrations of other programs through screen sharing, and various learning activities. We broadcast Visible Body 3D models (Figure 1A) and EdiErmes dissection clips to clarify concepts that are difficult to understand in 2D representations (Figure 1B).

### 2.3 Final tests of learning

The purpose of this study was to examine differences in learning human anatomy by comparing two groups of students: the first group had lectures and microanatomy laboratories F2F (Academic Year 2018/2019), the second theoretical lessons and microscopy experience online (Academic Years 2019/2020).

In both cases, the final examination consisted of the microscopic recognition of an anatomical structure and an oral examination on topographical and visceral anatomy topics. In detail, the subjects examined were: topographical anatomy of the head, neck, thorax, abdomen, pelvis, perineum, and limbs; and structural anatomy and morphology of the organs of the respiratory, digestive, urinary, female and male genitalia, endocrine, tegumentary, and cardiovascular systems. The oral examination was taken by students who had correctly recognized at least one of the two anatomical preparations observed under the microscope. It determined the final grade in thirtieths. Each student was evaluated by a commission comprising the teachers of the course, and the final grade was determined by the sum of the single evaluations (each teacher had 10 points available). The grade with honors (30/30 cum laude) was determined by unanimous consent among the teachers. Generally, the evaluations differed among teachers over a range from 0.3 to 1.4. The examining teachers were the same in both academic years. The final score is expressed in thirtieths. For both academic years, we compared the results obtained in seven different examination sessions (summer, autumn, and winter sessions).

In addition, to minimize the possibility of “suggestions” during the online examination, each student was framed by two cameras (computer and mobile phone).

Finally, we are aware that the reliability of oral examinations need not be poor, or at least no poorer than other assessment methods. We believe that increasing the number of oral examinations (about two to three students/hour) and the number of examiners improves the reliability (Daelmans et al., 2001; Johnson et al., 2018).

### 2.4 Evaluation by the students

Students in all Italian Universities can express their evaluation of the perceived quality of the didactics. For this purpose, 12 questions are used relating to the clarity of presentation, the availability of the teacher for further explanations, the need for basic preliminary knowledge to address the study better, and the perceived learning weight (Table 1). Here, we decided to offer a final question asking students to express their overall assessment of the course (“Are you overall satisfied about the didactic quality of the human anatomy course?”). All the responses were analyzed and grouped into “negative” and “positive.”

**TABLE 1:** Students' evaluation on the perceived quality of the didactics

	Strongly agree	Somewhat agree	Somewhat disagree	Strongly disagree
Was preliminary knowledge acquired sufficient for the understanding of the topics in the exam program?	4	3	2	1
Is the teaching load proportionate to the credits awarded?	4	3	2	1
Teaching materials (indicated and available) are suitable for the study of anatomy?	4	3	2	1
Have examination procedures been clearly defined?	4	3	2	1
Are schedules of theoretical lessons, practical activities, and other educational activities respected?	4	3	2	1
Does the teacher stimulate/motivate interest in the discipline?	4	3	2	1
Does teacher explain topics clearly?	4	3	2	1
Are additional educational activities (practical lessons, tutorials, workshops, etc.) useful for learning anatomy?	4	3	2	1
Was teaching carried out in a manner consistent with what was stated on the website of the course of study?	4	3	2	1
Are teachers available for clarifications and explanations?	4	3	2	1
Are you interested in the topics covered in this course?	4	3	2	1
Are you overall satisfied about the didactic quality of the human anatomy course?	4	3	2	1

### 2.5 Statistical analyses

Student's *t* test was used to compare the means between two groups, and nonparametric one‐way ANOVA (Kruskal–Wallis test) followed by the Dunn multiple comparisons test was used to compare the means among three or more groups. The median and the upper and lower limits of the 95% confidence interval for both the difference and the mean were also considered. The final score, 30 cum laude, was counted as 32 and then used in the statistical analysis.

The opinions of the students in both academic years were elicited using a short questionnaire. Items from a previous study (Attardi et al., 2018) were adapted for the present one (see Supplement). Responses were requested according to a four‐point Likert scale (4 = strongly agree, 3 = somewhat agree, 2 = somewhat disagree, and 1 = strongly disagree; Preedy, 2010). Nonparametric, one‐way ANOVA (Kruskal–Wallis test) followed by the Dunn multiple comparisons test was used to compare the data between the two groups because the scales in this survey were ordinal.

GraphPad Prism software 6.0 (GraphPad Software Inc., CA, USA) was used for all the analyses.

## 3 RESULTS

### 3.1 F2F versus online classes: Final examination scores comparison

Final examination scores were collected from students in two different academic years. Those of cohort 2018/2019 followed the course of Human Anatomy F2F, those of cohort 2019/2020 online. In each academic year, there were seven different examination sessions.

Among the students who took F2F lessons and laboratory experiences, 208 out of 249 successfully passed the examinations in the seven sessions, while 236 out of 248 students who took the lessons online passed the examination positively (*p* = 0.0001). The data are summarized in Table 2.

**TABLE 2:** Final scores (xx/30) of anatomy examinations per academic year

	Successful 2018/2019	Successful 2019/2020
Number of students	208	236
Minimum	18	18
25% Percentile	23	24
Median	25	27
75% Percentile	28	30
Maximum	30 cum laude	30 cum laude
Mean	25.33	26.69
Std. Deviation	3.581	3.625
Std. Error of Mean	0.2483	0.236

**FIGURE 2:**
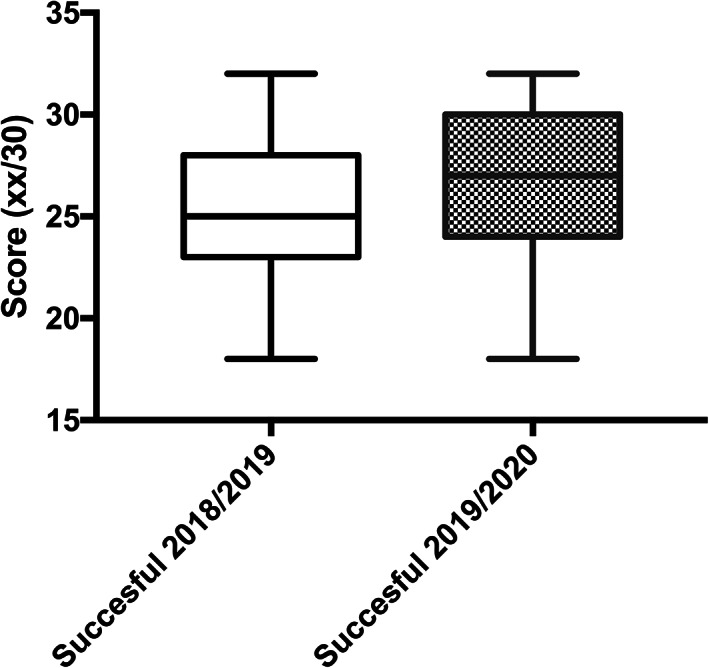
The analysis of the final score shows that the online group performed better

The final examination scores were analyzed (vote/30). Interestingly, the online group received a better final score (*p* = 0.0001). The results are summarized in Figure 2.

**FIGURE 3:**
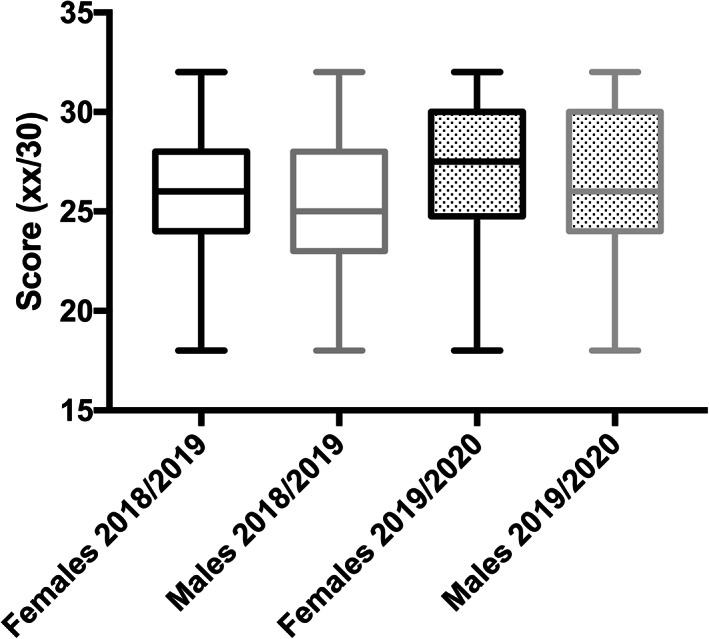
Analyses of differences in the final scores between sexes

We investigated possible differences in the final scores between sexes (Table 3). For females, 87.2% (143 out 164) who attended F2F lessons successfully passed the examination, compared to 95.2% (158 out 166) of those who attended online lessons. For males, the percentages of positive scores were 76.5% (65 out 85) and 95.1% (78 out 82), respectively. While there were no significant intra‐year differences, female students seemed to attain better scores than males (26 vs. 25 and 27.5 vs. 26, comparing the two teaching methods). Also, students who followed the online lessons obtained better final scores (*p* = 0.002 for females, and *p* = 0.0145 for males). For a summary see Figure 3.

**TABLE 3:** Differences in the final score (xx/30) of anatomy examinations by sex

	Females 2018/2019	Males 2018/2019	Females 2019/2020	Males 2019/2020
Number of values	143	65	158	78
Minimum	18	18	18	18
25% Percentile	24	23	24,75	24
Median	26	25	27.5	26
75% Percentile	28	28	30	30
Maximum	30 cum laude	30 cum laude	30 cum laude	30 cum laude
Mean	25.6	24.85	26.8	26.46
Std. Deviation	3.499	3.763	3.615	3.66
Std. Error of Mean	0.2926	0.4668	0.2876	0.4144
Lower 95% CI of mean	25.02	23.91	26.23	25.64
Upper 95% CI of mean	26.18	25.78	27.37	27.29

**FIGURE 4:**
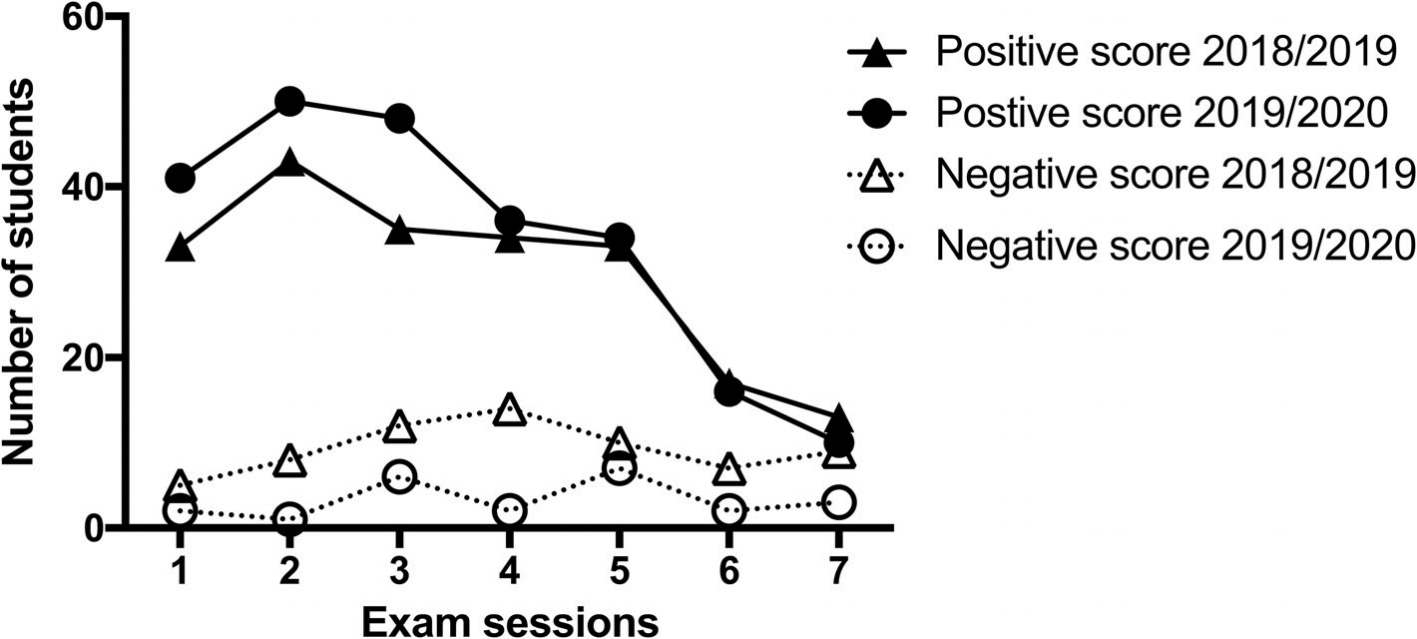
Analysis of changes in the positive and negative scores in each examination session

Analysis of the changes in positive and negative scores in each examination session revealed an interesting difference (Figure 4). In the first three examination sessions, the number of students who attended the online lessons achieved much better results than the F2F group. However, these differences decreased from the fourth to the seventh examination sessions. In contrast, the number of students who failed the examination increased among those who followed the F2F lessons.

**FIGURE 5:**
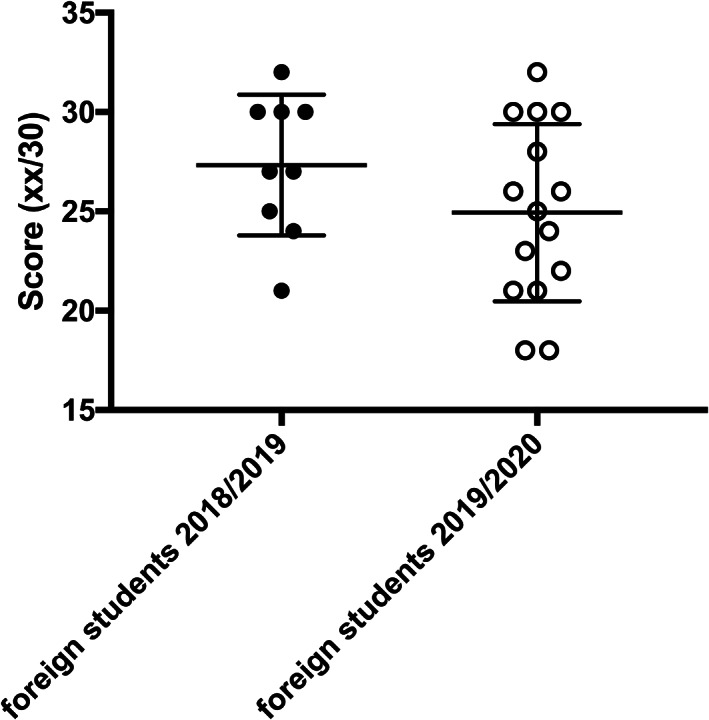
A different final score for foreign students is apparent, contrasting with what was seen among Italian students

A final point concerns foreign students. There were few of these, so the statistical value of the results is not strong. However, the final score shows an interesting result, contrasting with what we saw among Italian students (Figure 5). The number of students who passed the examination was higher among those who took online lessons (15 out 19 vs. 9 out 13); however, the result was better among foreign students who attended the F2F lessons (median score 27 vs. 25). Although the difference is not statistically significant (*p* = 0.2057), it could be related to the lower number of social relationships, and to the greater difficulty experienced by these students in overcoming the language barrier under conditions of social isolation.

### 3.2 Students' opinions on human anatomy F2F and online classes

**FIGURE 6:**
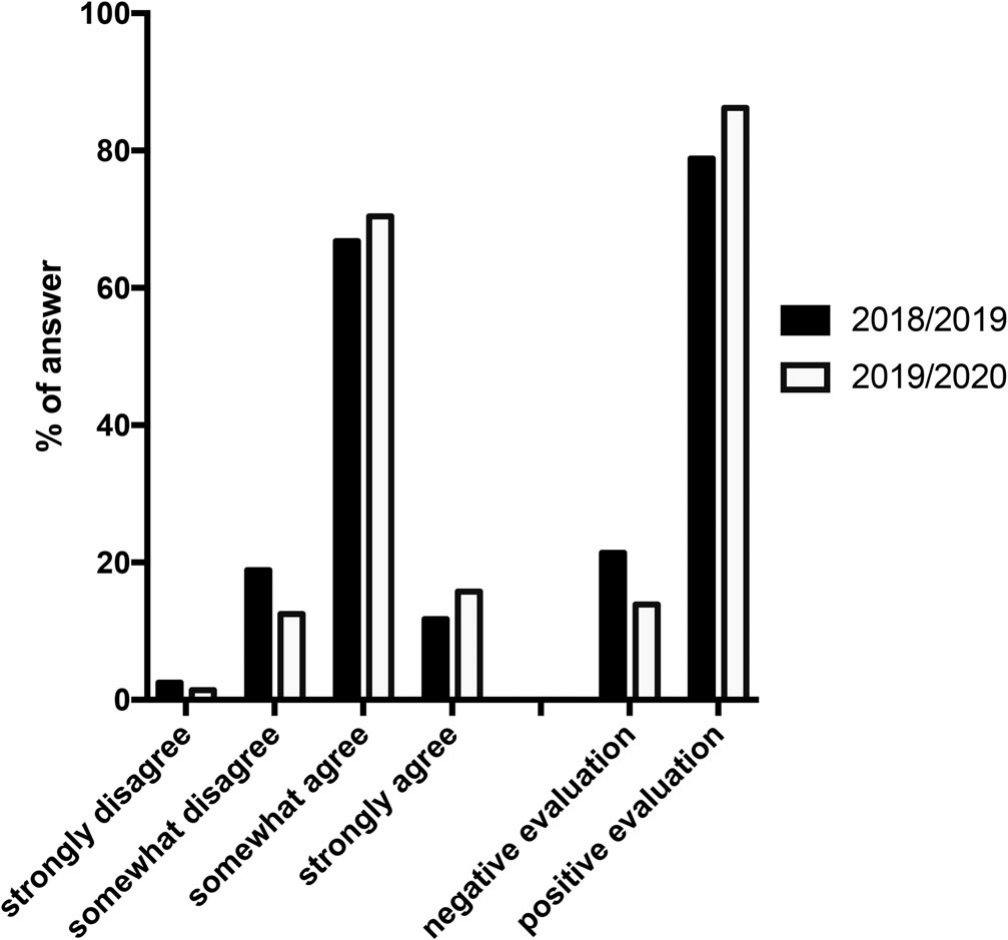
Students of all university courses in Italy can express their evaluation of the perceived quality of the didactics. Here, we decided to offer a final question asking students to express the overall assessment of the course (“Are you overall satisfied about the didactic quality of the human anatomy course?”). The two right columns represent the overall responses grouped into “negative” and “positive”

Our university regularly asks students to express their perception of the quality of teaching. Last year's students were fundamentally satisfied with the quality of the lessons and the interaction with teachers and among students through the online platform (Figure 6), in general even more than the F2F cohort. However, many students expressed dissatisfaction and suffering because of the lack of interpersonal interactions. The socializing aspects of F2F lessons, studying in libraries and/or study rooms, are important, and online lessons fall short from this point of view.

## 4 DISCUSSION

During the COVID‐19 pandemic, social distancing measures have isolated students from family and friends, so it could be more important than ever to provide a sense of community in universities and in medical schools. Although the evidence and experience concerning the best way to do this in anatomy education is limited and controversial (Franchi, 2020; Merrell, 2004; Srinivasan, 2020; Zhou et al., 2020), the pedagogy of distance learning can help us to design teaching methods to develop new online communities of anatomical investigation. Our teaching team has verified that the asynchronous and synchronous teaching association is a feasible approach, even for large cohorts of students (Green et al., 2014). The pedagogy of distance learning enables us to take advantage of the collaborative and interactive functions now available in online communication platforms. According to our experience, group‐learning activities can stimulate the attention of students and improve learning through webinars. However, a detailed survey will be required to assess the effect of virtual curricula on learning among medical school students.

The COVID‐19 pandemic and the resulting lockdown have led to changes in teaching methods to keep medical education active. Several authors have dealt with this, underlining the possible positive aspects (development of new online resources, exploitation of new technologies) and highlighting the opportunities (collaborations among teachers, distance work, incorporation of mixed learning into the future curriculum), and highlighting weaknesses (time constraints, lack of practical sessions and dissection of the corpse, problems with evaluation) and threats (reduced student involvement, decreased student/student and teacher/student ratios; Longhurst et al., 2020). Finally, several authors have strongly stressed the need for communication technologies such as video conferencing, social media, and network platforms (Moszkowicz et al., 2020).

Trying to summarize messages from all these articles, the usefulness of online learning methods is undisputed (Evans et al., 2020; Flynn et al., 2021) and should supplement innovative and effective medical learning (Flynn et al., 2021; Iwanaga et al., 2021; Longhurst et al., 2020; Moszkowicz et al., 2020; Pacheco et al., 2020; Pather et al., 2020; Singal et al., 2020). If these new methods are implemented, they will constitute very useful novel‐learning resources in the future post COVID‐19 period when we will return mainly to F2F classes; they will enable us to avoid losing the skills that have been learned in this period; but above all, the integrated approach will allow learning to be customized, aiming to achieve deeper and more student‐centered learning (Lampugnani, 2020).

Analysis of the students' final grades in the anatomy examinations shows that examinations taken at the end of the online learning course had a higher average than those obtained at the end of the F2F course. In addition, more students obtained positive results in the summer session than in the autumn and winter sessions. These positive results demonstrate that students in the University of Genoa during the period of lockdown during the second semester of the course had good opportunities for learning through the didactics distributed with the online modality. It is possible that the students who followed the online lessons benefited from better access to educational activities and materials. In particular, the possibility of listening several times to the recorded lessons seems to be relevant. However, it is also possible that not wasting time in moving from home to university, and the inability to go out and participate in social activities, increased the time spent in studying. This could have influenced the preparation and therefore the final result positively. However, this period of lockdown has not been qualitatively good in all respects. In fact, students have often complained of psychophysical stress, which in some cases could have made it difficult to achieve the concentration necessary for studying.

In addition, we noticed that students engaged discreetly in discussion, sometimes even better than we had previously experienced in F2F teaching (Flynn et al., 2021; Johnson et al., 2013; McBrien et al., 2009). One reason could be that students asked questions anonymously and perhaps felt more comfortable than during F2F lessons. Thus, some students felt more confident in virtual interactions than physical environments (Flynn et al., 2021; McBrien et al., 2009; Murphy & Rodríguez Manzanares, 2008).

Foreign students were in a different situation. They suffered the most from social distancing. In fact, even if their numbers were not sufficient for a statistical survey, it is evident that those who followed the online lessons obtained worse examination results. There could be several reasons. These students had arrived in Italy recently, and the subsequent lockdown forced them to have little contact with other students and also with the teachers. As a result, learning the Italian language could have been impaired, and this could have undermined their confidence in the final examination.

Were the higher grades in the summer session due to more permissive behavior among teachers in carrying out distance examinations than face‐to‐face ones? This is possible; however, it should be noted that the same teachers presented the lessons and examinations in the two different academic years, and they used the same methods of judgment regardless of the mode of lesson and examination.

The aim of this study is to show that F2F and online teaching methods are not merely interchangeable but can offer educational benefits. Our purpose as anatomy teachers should be to build customizable and student‐centered courses. In fact, the different methods could have different effects on individual students depending on their needs. Students with mobility difficulties, students who live away from campus or international students could benefit from a mixed system combining F2F lessons with synchronous or asynchronous online lessons. In addition, the recorded and easily accessible online lessons could allow students to follow insights gained in the classroom, preferably using innovative methods such as 3D human body reconstruction and/or virtual reality. For this, students could be divided into small groups during the in‐depth lessons. Obviously, the laboratory activities of both topographic anatomy using 3D systems and virtual reality and microanatomy under the microscope should preferably remain in the classroom. Further studies could explore students' perceptions of this mixed educational method to organize the future of learning anatomy.

In conclusion, we consider it necessary to reorganize teaching in medical schools even when the pandemic is finally resolved. Lessons need to be implemented using different teaching techniques complementary to conventional F2F education. Experience at the University of Genoa shows that distance learning in the teaching of human anatomy was perceived as useful and positive by most students. Distance learning can be an effective support for anatomy teaching, facilitating a different mode of learning in which lessons and study are more sensitive to the individual's schedule and needs. Of course, we should not and cannot exclude face‐to‐face teaching.
